# A therapeutic preconceptional vaccine against Chagas disease: A novel indication that could reduce congenital transmission and accelerate vaccine development

**DOI:** 10.1371/journal.pntd.0006985

**Published:** 2019-01-31

**Authors:** Eric Dumonteil, Claudia Herrera, Pierre Buekens

**Affiliations:** 1 Department of Tropical Medicine, School of Public Health and Tropical Medicine, and Vector Borne and Infectious Disease Research Center, Tulane University, New Orleans, Louisiana, United States of America; 2 Department of Epidemiology, School of Public Health and Tropical Medicine, Tulane University, New Orleans, Louisiana, United States of America; National Institutes of Health, UNITED STATES

Chagas disease, or American trypanosomiasis, is caused by the protozoan parasite *Trypanosoma cruzi*. It is a major cause of cardiac disease in the Americas. At least 6 million people are currently infected in Latin America, including 1 million women of reproductive age [1]. In the United States, the estimated number of *T*. *cruzi*-infected women of reproductive age was 130,522 in 2000 [2]. Healthcare costs of the disease amount to over US$24 billion [3]. Treatment options for infected patients are limited, with only two drugs available (Benznidazole and Nifurtimox), and require prolonged treatments, which have limited efficacy and important adverse effects [4]. New treatments are therefore needed to improve patient care [5].

## Congenital transmission of *T*. *cruzi*

Mothers can transmit *T*. *cruzi* to their babies during pregnancy, and infected babies are at risk of developing chronic Chagas disease later in life [6]. A meta-analysis of published data showed a 5.0% congenital transmission rate in endemic countries [7]. There is a risk of pregnancy complications, including preterm premature rupture of membranes and preterm delivery. *T*. *cruzi*-infected newborns may have severe morbidity and are at risk of neonatal intensive care unit hospitalization and neonatal mortality [8]. Available drugs are not approved for use during pregnancy. Asymptomatic infected newborns can be effectively treated if detected early, but a follow-up to at least eight months of age is needed in most cases to diagnose congenital transmission by measuring persisting antibodies [6]. Losses to follow-up are frequent, and many infected infants remain untreated. There is therefore an urgent need to prevent congenital transmission of *T*. *cruzi*.

## Preconceptional benznidazole treatment to reduce *T*. *cruzi* congenital transmission

Several retrospective observational studies suggest that infected women treated at a young age do not transmit *T*. *cruzi* when pregnant later in life [9–13]. The first study included 32 children born to 16 women who were treated with benznidazole when they were 6 to 15 years old and who were evaluated 14 years later [10]. None of the children were infected. A larger observational study compared women treated before pregnancy to untreated women [9]. On average, women were treated 17 years before follow-up. Among the 222 children born to untreated women, 34 were infected with *T*. *cruzi* (15.3%), whereas no infection was found among the 132 children of previously treated women. Another small observational study found no congenital transmission among 15 women who became pregnant from 1 to 8 years after treatment [11]. More recent studies also point out the absence of congenital transmission from infected mothers previously treated with benznidazole [12, 13]. Although no randomized controlled trial is available, those observational studies suggest that reducing maternal parasitemia before conception reduces the risk of congenital transmission.

Expert consensus recommends that *T*. *cruzi* seropositive women of reproductive age should be treated [10, 14]. However, the fear of side effects limits the implementation of benznidazole treatment [15]. Indeed, current doses of benznidazole can cause dermatitis, which usually occurs during the first weeks, and peripheral neuropathy, which seems to be related to the cumulative dose and may take months to resolve [16–18]. Gastrointestinal effects, including vomiting and pain, are also frequent side effects that can be theoretically prevented by diet. Other severe adverse effects, although infrequent, are bone marrow depression, toxic hepatitis, and lymphomas [18]. The Benznidazole Evaluation for Interrupting Trypanosomiasis (BENEFIT) trial compared benznidazole versus placebo among patients with Chagasic cardiomyopathy and is the largest placebo-controlled randomized trial performed thus far [19]. Of concern, the rate of treatment interruption because of an adverse event was 23.9% in the benznidazole group compared to 9.5% in the placebo group, and 13.4% of patients in the benznidazole group permanently discontinued treatment compared to 3.6% in the control group. Dermatitis, digestive intolerance, and neuropathy accounted for more than 90% of the interruptions [20]. Therefore, although preconceptional treatment appears very promising, the frequency of side effects limits its use and alternative approaches to reduce parasitemia before conception should be investigated. These include treatments with reduced doses and/or shorter regimens, combination therapies, and therapeutic vaccination for the prevention of congenital transmission.

## Development of a Chagas disease therapeutic vaccine

Therapeutic vaccination has been proposed for the control of *T*. *cruzi* infection, either as a stand-alone immunotherapeutic tool or in combination with antiparasitic treatment [21]. The initial target product profile is a vaccine to stop or at least delay the progression of cardiac complications in infected patients [21]. In combination with drug therapy, the vaccine may allow lowering drug dose and/or duration of treatment, which may increase the tolerability of the drug and reduce its adverse side effects. After many years of debate on the role of autoimmunity in triggering Chagas disease progression, which considerably limited the efforts at developing a vaccine, it is now well established that parasite persistence in tissues is the main driving mechanism of pathogenesis. This provides a strong rationale for vaccine development [21]. Extensive preclinical studies using a variety of vaccine formulations—such as live-attenuated parasites, recombinant proteins, DNA or viral vectors with a diverse set of adjuvants and carriers ranging from cytokines, TLR agonists or nanoparticles—have evidenced the ability of some vaccine formulations to control *T*. *cruzi* infection in mouse models [22–24]. Some of these vaccine candidates have been tested as preventative vaccines, others as therapeutic vaccines, that are able to redirect the immune response to increase its efficacy at controlling the parasite in an infected host. In particular, the ability of several vaccine formulations to reduce parasitemia and parasite burden in cardiac tissue of infected animals is well established (Table 1). These studies serve as a proof of concept and rationale for the feasibility of a vaccine against *T*. *cruzi*. Based on these premises, a public–private consortium has been established to pursue the development of a therapeutic vaccine against Chagas disease [21].

**Table 1:** Selected *T*. *cruzi* vaccine candidates.

Vaccine type	Formulation	Reduction in parasitemia	Reference
Recombinant proteins	Tc24 and TSA-1 with Th1 adjuvant	Yes	[21, 25, 26]
Recombinant proteins	TS with diverse adjuvants	Yes	[27]
Recombinant protein	Tc80 oligopeptidase with CpG adjuvant	Yes	[28]
Recombinant proteins/DNA vaccine	TcG2/TcG4 DNA prime/protein boost with cytokine adjuvants	Yes	[29]
Recombinant adenovirus	Adenovirus expressing ASP-2 and TS	Yes	[23]
Live attenuated parasites	Specific gene deletion causing attenuation	Yes	[30]

**Abbreviations**: ASP-2, Amastigote surface protein; Tc24, *T*. *cruzi* 24 kDa antigen; Tc80, *T*. *cruzi* 80 kDa antigen; TcG2/TcG4, *T*. *cruzi* G2/G4 antigens; Th1, T helper 1; TS, *trans*-sialidase; TSA-1, Trypomastigote surface antigen.

## Towards a preconceptional therapeutic vaccine to prevent congenital transmission of *T*. *cruzi*

A preconceptional vaccine aimed at preventing future congenital transmission of the parasite would be an excellent additional tool for Chagas disease control and may be the basis of a novel target product profile. Because it is generally accepted that mother parasitemia is a key factor modulating congenital transmission [31, 32], this provides a fast and easy end point for the clinical evaluation of a therapeutic preconceptional vaccine, which may focus on a decrease in mother parasitemia. This would greatly shorten the follow-up required for an initial assessment of vaccine efficacy in clinical trials. Women of reproductive age can also be expected to be a rather healthy population, mostly asymptomatic or in the earlier stages of Chagasic cardiomyopathy, thus corresponding to a more homogenous population than that of the BENEFIT trial, which may allow reducing the size of a vaccine trial without compromising its power.

Preclinical studies of a preconceptional *T*. *cruzi* vaccine in infected animals should therefore be conducted to explore the feasibility of such a vaccine. Several rodent models of *T*. *cruzi* congenital transmission have been described but may have limited relevance for congenital transmission in humans due to variability in the timing of infection and pregnancies, as well as placental differences [33, 34]. Nonetheless, studies in rodent models may help evaluate possible sex-specific responses to a *T*. *cruzi* vaccine, as recommended by current National Institutes of Health (NIH) policy. Preclinical studies of a preconceptional vaccine in nonhuman primates may also be warranted to account for the unique features of human and/or primate placenta [33, 34] and their role modulating the transmission of *T*. *cruzi* parasites. The available nonhuman primate models of experimental *T*. *cruzi* infection, as well as the existence of naturally infected animals in many nonhuman primate facilities, represent valuable opportunities for such studies [35, 36].

Although reaching Chagas disease control and vaccine development will require strong investments, the economic benefits to individuals and society far exceed these investments. Developing a preconceptional therapeutic vaccine may provide a unique opportunity to accelerate vaccine evaluation in clinical trials, as well as provide a novel alternative for the control of congenital transmission of *T*. *cruzi*.
